# Health Care Utilization During the COVID-19 Pandemic Among Individuals Born Preterm

**DOI:** 10.1001/jamanetworkopen.2023.10696

**Published:** 2023-04-28

**Authors:** Elisabeth C. McGowan, Monica McGrath, Andrew Law, T. Michael O’Shea, Judy L. Aschner, Courtney K. Blackwell, Rebecca C. Fry, Jody M. Ganiban, Rosemary Higgins, Amy Margolis, Sheela Sathyanarayana, Genevieve Taylor, Akram N. Alshawabkeh, José F. Cordero, Nicole T. Spillane, Mark L. Hudak, Carlos A. Camargo, Dana Dabelea, Anne L. Dunlop, Amy J. Elliott, Assiamira M. Ferrara, Maria Talavera-Barber, Anne Marie Singh, Margaret R. Karagas, Catherine Karr, Thomas G. O’Connor, Nigel Paneth, Rosalind J. Wright, Robert O. Wright, Whitney Cowell, Joseph B. Stanford, Casper Bendixsen, Barry M. Lester

**Affiliations:** 1 The Warren Alpert School of Medicine, Brown University, Providence, Rhode Island; 2 Johns Hopkins Bloomberg School of Public Health, Baltimore, Maryland; 3 The University of North Carolina at Chapel Hill, Chapel Hill; 4 Albert Einstein College of Medicine, New York, New York; 5 Hackensack University Medical Center and Hackensack Meridian School of Medicine, Hackensack, New Jersey; 6 Northwestern University Feinberg School of Medicine, Chicago, Illinois; 7 George Washington University, Washington, District of Columbia; 8 Department of Global and Community Health, College of Health and Human Sciences, George Mason University, Fort Myers, Florida; 9 Marieb College of Health and Human Services, Florida Gulf Coast University, Fort Myers; 10 Columbia University Irving Medical Center, New York, New York; 11 University of Washington, Seattle; 12 Seattle Children’s Research Institute, Seattle, Washington; 13 Northeastern University, Boston, Massachusetts; 14 Department of Epidemiology and Biostatistics, University of Georgia, Athens; 15 Department of Pediatrics, University of Florida College of Medicine, Jacksonville; 16 Massachusetts General Hospital, Boston; 17 Lifecourse Epidemiology of Adiposity and Diabetes Center, University of Colorado Anschutz Medical Campus, Aurora; 18 Emory University, Atlanta, Georgia; 19 Avera Research Institute, Sioux Falls, South Dakota; 20 Kaiser Permanente Northern California Division of Research, Oakland; 21 Avera McKennan Hospital, Sioux Falls, South Dakota; 22 University Health Center, Avera Research Institute, Sioux Falls, South Dakota; 23 Department of Pediatrics, University of Wisconsin School of Medicine and Public Health, Madison; 24 Geisel School of Medicine at Dartmouth, Lebanon, New Hampshire; 25 Department of Environmental and Occupational Health Sciences, University of Washington, Seattle; 26 University of Rochester Medical Center, Rochester, New York; 27 Department of Epidemiology and Biostatistics, College of Human Medicine, Michigan State University, East Lansing; 28 Department of Food Science and Human Nutrition, Michigan State University, East Lansing; 29 Institute for Exposomic Research, Icahn School of Medicine Mount Sinai, New York, New York; 30 Department of Pediatrics, NYU Grossman School of Medicine, New York, New York; 31 University of Utah, Salt Lake City; 32 Marshfield Clinic Research Institute, Marshfield Clinic Health System, Marshfield, Wisconsin

## Abstract

**Importance:**

Limited data exist on pediatric health care utilization during the COVID-19 pandemic among children and young adults born preterm.

**Objective:**

To investigate differences in health care use related to COVID-19 concerns during the pandemic among children and young adults born preterm vs those born at term.

**Design, Setting, and Participants:**

In this cohort study, questionnaires regarding COVID-19 and health care utilization were completed by 1691 mother-offspring pairs from 42 pediatric cohorts in the National Institutes of Health Environmental Influences on Child Health Outcomes Program. Children and young adults (ages 1-18 years) in these analyses were born between 2003 and 2021. Data were recorded by the August 31, 2021, data-lock date and were analyzed between October 2021 and October 2022.

**Exposures:**

Premature birth (<37 weeks’ gestation).

**Main Outcomes and Measures:**

The main outcome was health care utilization related to COVID-19 concerns (hospitalization, in-person clinic or emergency department visit, phone or telehealth evaluations). Individuals born preterm vs term (≥37 weeks’ gestation) and differences among preterm subgroups of individuals (<28 weeks’, 28-36 weeks’ vs ≥37 weeks’ gestation) were assessed. Generalized estimating equations assessed population odds for health care used and related symptoms, controlling for maternal age, education, and psychiatric disorder; offspring history of bronchopulmonary dysplasia (BPD) or asthma; and timing and age at COVID-19 questionnaire completion.

**Results:**

Data from 1691 children and young adults were analyzed; among 270 individuals born preterm, the mean (SD) age at survey completion was 8.8 (4.4) years, 151 (55.9%) were male, and 193 (71.5%) had a history of BPD or asthma diagnosis. Among 1421 comparison individuals with term birth, the mean (SD) age at survey completion was 8.4 (2.4) years, 749 (52.7%) were male, and 233 (16.4%) had a history of BPD or asthma. Preterm subgroups included 159 individuals (58.5%) born at less than 28 weeks’ gestation. In adjusted analyses, individuals born preterm had a significantly higher odds of health care utilization related to COVID-19 concerns (adjusted odds ratio [aOR], 1.70; 95% CI, 1.21-2.38) compared with term-born individuals; similar differences were also seen for the subgroup of individuals born at less than 28 weeks’ gestation (aOR, 2.15; 95% CI, 1.40-3.29). Maternal history of a psychiatric disorder was a significant covariate associated with health care utilization for all individuals (aOR, 1.44; 95% CI, 1.17-1.78).

**Conclusions and Relevance:**

These findings suggest that during the COVID-19 pandemic, children and young adults born preterm were more likely to have used health care related to COVID-19 concerns compared with their term-born peers, independent of a history of BPD or asthma. Further exploration of factors associated with COVID-19–related health care use may facilitate refinement of care models.

## Key Points

**Question:**

Are there differences in health care use during the COVID-19 pandemic among children and young adults (ages 1-18 years) who were born preterm vs those born term?

**Findings:**

In this cohort study, data from 1691 mother-offspring pairs were collected on health care utilization related to COVID-19 concerns. Individuals who were born at less than 28 weeks’ gestation had twice the odds of either in-person or phone or telehealth evaluations during the COVID-19 pandemic compared with those born at term.

**Meaning:**

The findings of this cohort study may help broaden understanding factors driving health care utilization during pandemic surges and facilitate refinement and flexibility of care models.

## Teaser

This cohort study investigates differences in health care use related to COVID-19 concerns during the pandemic among individuals born preterm vs those born at term.

## Introduction

The COVID-19 pandemic has caused waves of public health crises. Among the pediatric population, data are now emerging on clinical characteristics of children with severe COVID-19,^1^ risk factors associated with COVID-19,^2,3,4^ and the effects of COVID-19 on health care utilization.^5,6,7,8,9^ Individuals born preterm (ie, <37 weeks’ gestation) are in a distinct risk group, with medical fragility and special needs, including increased rates of diseases, such as bronchopulmonary dysplasia (BPD).^10^ A 2022 meta-analysis that included only 2 studies of preterm infants reported that the risk of severe COVID-19 was 2 times higher in neonates born preterm than in those born at term.^11^

Pandemic-related surges created shifts in health care use, including overall reductions in emergency department (ED),^12^ subspecialty,^9^ and primary care visits,^7^ along with rapid increases in telehealth visits. Among children, several comorbidities have been associated with COVID-19–related health care utilization, including lung, cardiac, neurologic, and neurodevelopmental disorders.^11^ More than 10% of all births in the US are preterm^13^ and are associated with billions of dollars in estimated costs^14^; thus, it is worthwhile to explore pandemic-related health care utilization patterns that were prompted by COVID-19 concerns among individuals born preterm.

Health care facilities can benefit from a deeper understanding of factors that influence resource utilization to ensure that the flexibility and refinement of models can efficiently adjust to pandemic surges. Yet, there is a significant lack of data on health care utilization during the COVID-19 pandemic among individuals born preterm. Leveraging the National Institutes of Health (NIH)–funded Environmental Influences on Child Health Outcomes (ECHO) Program provides an opportunity to explore health care use during the COVID-19 pandemic among a large sample of children and young adults ages 1 to 18 years. Our primary objective was to investigate health care utilization differences between individuals who were born preterm (<37 weeks’ gestation) compared with those born at term (≥37 weeks’ gestation). We further examined the association of being born extremely preterm (<28 weeks’ gestation) with health care utilization. We hypothesized that individuals born preterm and extremely preterm had increased utilization of care related to COVID-19 concerns compared with individuals born at term.

## Methods

This cohort study was approved by local and central ECHO institutional review boards. Written informed consent was obtained for participation in specific cohorts and the ECHO-wide Cohort Data Collection Protocol. This study is reported following the Strengthening the Reporting of Observational Studies in Epidemiology (STROBE) reporting guideline.

### Participants

The ECHO Program was designed to investigate the effects of early life exposures on 5 areas of child health: prenatal, perinatal, and postnatal health; obesity; respiratory conditions; neurodevelopment; and positive health and well-being.^15,16^ The ECHO Program’s goal is to leverage existing US pediatric studies by combining data collected via cohort-specific protocols, with data collected via a standardized ECHO-wide protocol.^17,18^

### Study Population

**Figure 1.**
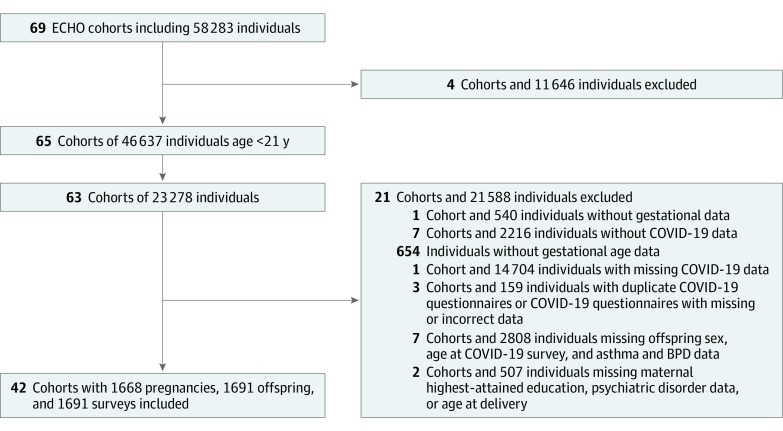
Flowchart of Study Participants and Survey Selection

To investigate preterm birth and differences in health care utilization and symptoms during the COVID-19 pandemic, we restricted our study population to mother-offspring pairs with complete data for the following variables of interest: gestational age (GA) at birth, singleton vs multiple birth, COVID-19 outcomes of interest, maternal age at delivery, offspring age at COVID-19 questionnaire administration, maternal education, maternal race and ethnicity, offspring sex, maternal history of psychiatric disorders, and information on respiratory disease (offspring BPD or asthma). Covariates were selected based on consistency with research findings and hypothesized associations with exposure and outcome.^19,20,21,22^ Children and young adults ages 1 to 18 years were included. Figure 1 provides a study population flowchart. Six ECHO cohorts consisted of participants born very preterm (<32 weeks’ gestation).^23^ After excluding cohorts and participants with missing key data, the final analytical data set included 42 cohorts of 1691 mother-offspring pairs, including 270 individuals born preterm and 1421 individuals born at term.

### Sociodemographic Variables

Maternal educational status was grouped into 3 categories based on the highest level of attainment: less than high school or a high school graduate, some college with no degree or an associate’s degree, and a bachelor’s degree and above. Offspring and maternal race and ethnicity were caregiver-reported and self-reported, respectively, and categorized as American Indian or Alaska Native, Asian, Black, Native Hawaiian or Pacific Islander, White, multiple races, and other race. For analyses, race and ethnicity categories were collapsed due to small sample sizes and included Hispanic, non-Hispanic Black, non-Hispanic White, and non-Hispanic other race. The non-Hispanic other race category included those who were non-Hispanic and Alaska Native, American Indian, Asian, Native Hawaiian, or Pacific Islander or identified as multiple races.

### Maternal Medical History

A history of maternal psychiatric disorder was defined as any diagnosis of a psychiatric disorder, including major depression, dysthymia, bipolar disorder, anxiety disorder, specific phobia, panic disorder, obsessive compulsive disorder, social anxiety, posttraumatic stress disorder, and attention-deficit/hyperactivity disorder. Data were derived from medical records, self-report, and *International Classification of Diseases, Ninth Revision *(*ICD-9*) codes (ie, 296.2, 296.3, 296.9, 300.0, 300.2, 300.3, 300.4, 309.2, 309.8) or *International Statistical Classification of Diseases and Related Health Problems, Tenth Revision * (*ICD-10*) (depression and anxiety only: F32-F34, F39, 40.0-40.2, 40.8, 40.9, 41.0, 41.1, 41.8, 41.9, 42, 43.1, 93.0).

### Offspring Medical History

GA at birth was obtained through maternal and offspring medical records or parent report. Preterm birth was defined as birth at less than 37 weeks’ gestation, and term birth was defined as birth at 37 weeks’ gestation or more. Subgroups of preterm birth included individuals born at less than 28 weeks’ gestation (extremely preterm), 28 to 31 weeks’ gestation (very preterm), 32 to 33 weeks’ gestation (moderate preterm), and 34 to 36 weeks’ gestation (late preterm). A BPD diagnosis was defined by the NIH,^24^ room-air challenge test,^25^ or the criteria described by Shennan et al.^26^ Participants missing BPD information and born at 37 weeks’ gestation or more were assumed to not have a BPD diagnosis. Asthma diagnosis was provided by parent or caregiver report of a physician or health care practitioner having diagnosed the offspring with asthma up to the current visit. Offspring sex was the designated sex at birth (male or female).

### ECHO COVID-19 Questionnaire

The ECHO Program developed primary caregiver–reported COVID-19 questionnaires in April 2020 to assess pandemic-related changes to the caregiver’s and offspring’s health, associations with health care utilization and behaviors, as well as changes in lifestyle behaviors and stress responses. Caregivers were asked to respond based on the impact of the COVID-19 pandemic on the ECHO-enrolled individual. Overall, 98% of the data were reported by biological mothers. Questionnaires were completed between April 2020 and August 2021 (eFigure in Supplement 1), and the first questionnaire was analyzed if multiple questionnaires were completed.

Our primary outcome of health care utilization related to COVID-19 symptom concerns was defined as an overnight hospital stay because a practitioner suspected that the individual had SARS-CoV-2 virus infection; the individual sought care in-person at a clinic, physician’s office, urgent care, or ED related to symptoms concerning for COVID-19; or a parent contacted a practitioner over the phone, by email, or online regarding concerns for COVID-19. COVID-19–related symptoms prompting health care utilization included any of the following: respiratory symptoms (cough, shortness of breath, runny nose), fever, headache, muscle aches, fatigue, itchy eyes, gastrointestinal symptoms (diarrhea, nausea, vomiting), and loss of smell or taste. A secondary outcome of overall health care utilization changes during the COVID-19 pandemic was defined as missed health care appointments either related to parental concerns about entering a practitioner office or practitioner cancellations.

### Statistical Analysis

Maternal sociodemographic characteristics and maternal and offspring medical history for individuals born preterm and term are presented as means and SDs for continuous variables. The number of observations and percentage of total observations are presented for categorical variables. We performed χ^2^ and *t* tests for categorical and continuous variables, respectively. We used generalized estimating equations (GEE) to account for the correlation structure of health care utilization related to COVID-19 concerns within the cohort.^27,28^ COVID-19–related outcomes of interest were binary; therefore, GEE analyses were used with a binomial distribution and logit link. We used robust SEs and an exchangeable correlation matrix. Unadjusted and adjusted GEE were performed to calculate a population odds ratio (OR) and 95% CI. The exposure variable of GA at birth was modeled as a binary variable (preterm <37 weeks’ vs term ≥37 weeks’ gestation: model 1) and as a categorical variable (<28 weeks’, 28-36 weeks’, and ≥37 weeks’ gestation: model 2). All models were adjusted for month and year of COVID-19 questionnaire completion, offspring age at questionnaire completion time, and risk factors previously associated with health care utilization or negative health outcomes, including maternal age (centered at 31 years of age [median]), singleton vs multiple birth, offspring sex, offspring diagnosis of asthma or BPD, maternal education, and a maternal history of a psychiatric disorder, as well as ECHO cohort membership. Maternal race and ethnicity data were combined and adjusted for in the analyses. A 2-sided *P* < .05 was taken as a statistically significant association. These analyses were conducted using R version 3.6.3 (R Project for Statistical Computing). The GEE analyses were performed using geeglm function from the “geekpack” package.^29,30,31^ Data were analyzed from October 2021 to October 2022.

## Results

### Study Participant Characteristics

Data from 1691 children and young adults were analyzed, including 270 individuals born preterm (mean [SD] age at questionnaire completion, 8.8 [4.4] years; 151 [55.9%] male) and 1421 individuals born at term (mean [SD] age at questionnaire completion, 8.4 [2.4] years; 749 [52.7%] male) (Table 1). The distribution of offspring age at COVID-19 questionnaire completion was 293 questionnaires (17%) in early childhood (age 1-5 years), 1297 questionnaires (77%) in middle childhood (6-11 years), and 97 (6%) for adolescence (12-18 years).

**Table 1.** Descriptive Characteristics of Cohort

Characteristic	Individuals, No. (%)		P value
	Born preterm (<37 wk) (n = 270)	Born term (≥37 wk) (n = 1421)	
Gestational age, wk			
Mean (SD)	27.9 (3.6)	39.2 (1.2)	<.001
<28 (extremely preterm)	158 (58.5)	NA	NA
28-31 (very preterm)	67 (24.8)	NA	NA
32-33 (moderate preterm)	6 (2.2)	NA	NA
34-36 (late preterm)	39 (14.4)	NA	NA
≥37 (term)	NA	1421 (100)	NA
Singleton gestation	207 (76.7)	1398 (98.4)	<.001
Maternal age at delivery, mean (SD), y	30.5 (6)	30.4 (5)	<.001
Maternal race and ethnicity			
Hispanic	32 (12.1)	126 (8.9)	<.001
Non-Hispanic Asian	12 (4.5)	35 (2.5)	
Non-Hispanic Black	35 (13.2)	95 (6.7)	
Non-Hispanic other Race^a^	23 (8.7)	95 (6.7)	
Non-Hispanic White	163 (61.5)	1064 (75.2)	
Maternal race			
American Indian or Alaska Native	<5 (<3)^b^	41 (2.9)	
Asian	12 (4.6)	35 (2.5)	
Black	38 (14.5)	106 (7.5)	
Native Hawaiian or other Pacific Islander	0	<5 (<1)^b^	
White	<170 (<67)^b^	<1140 (<82)^b^	
Multiple	26 (9.9)	63 (4.5)	
Other	8 (3.0)	22 (1.6)	
Missing	8 (3.0)	13 (0.9)	
Maternal ethnicity			
Hispanic	32 (12.0)	126 (8.9)	.15
Non-Hispanic	235 (88.0)	1290 (91.1)	
Highest maternal education			
≤High school	43 (15.9)	124 (8.7)	<.001
Some college (no degree) or associate’s degree	80 (29.6)	347 (24.4)	
≥Bachelor’s degree	147 (54.4)	950 (66.9)	
History of maternal psychiatric disorder	133 (49.3)	641 (45.1)	.21
Offspring race and ethnicity			
Hispanic	41 (15.2)	182 (12.8)	<.001
Non-Hispanic Asian	8 (3.0)	26 (1.8)	
Non-Hispanic Black	33 (12.2)	91 (6.4)	
Non-Hispanic White	153 (56.7)	984 (69.3)	
Non-Hispanic other race^a^	35 (13.0)	26 (1.8)	
Offspring race			
American Indian or Alaska Native	<5 (<1.0)^b^	<5 (<1.0)^b^	<.001
Asian	9 (3.4)	28 (2.0)	
Black	35 (13.1)	103 (7.3)	
Native Hawaiian or other Pacific Islander	0	<5 (<1.0)^b^	
White	<180 (<66)^b^	<1090 (<78)^b^	
Multiple	41 (15.3)	134 (9.5)	
Other	6 (2.2)	10 (0.7)	.36
Offspring sex			
Male	151 (55.9)	749 (52.7)	.35
Female	119 (44.1)	672 (47.3)	
Offspring age at COVID-19 survey completion, mean (SD), y	8.8 (4.4)	8.4 (2.4)	<.001
Offspring medical risk, No./No. with available data (%)			
BPD	121/229 (52.8)	0	<.001
Asthma	116/265 (43.7)	233/1421 (16.4)	<.001
BPD and/or asthma	193/270 (71.5)	233/1421 (16.4)	<.001
Wheezing or whistling in chest over past 12 mos	114/262 (43.5)	291/1405 (20.7)	<.001

Abbreviations: BPD, bronchopulmonary dysplasia; NA, not applicable.

^a^ The Non-Hispanic Other Race category included those who were Non-Hispanic and Alaska Native, American Indian, Asian, Native Hawaiian, or Pacific Islander, and multiple races.

^b^ In accordance with the publication and data use policy of the Environmental Influences on Child Health Outcomes program, cells with fewer than 5 individuals are suppressed for privacy. The sample size in additional cells are also suppressed to prevent calculation of the exact sample size in cell with fewer than 5 participants.

The mean (SD) GA of participants born preterm was 27.9 (3.6) weeks, with most (158 individuals [58.5%]) born at less than 28 weeks’ gestation. Mothers of individuals born preterm were more likely to have been of younger age at delivery, have a multiple-gestation birth, and have less educational attainment compared with mothers of term-born individuals (Table 1). Maternal race and ethnicity varied significantly between preterm and term birth groups (32 Hispanic mothers [12.1%] vs 126 Hispanic mothers [8.9%]; 12 non-Hispanic Asian mothers [4.5%] vs 35 non-Hispanic Asian mothers [2.5%]; 35 non-Hispanic Black mothers [13.2%] vs 95 non-Hispanic Black mothers [6.7%]; 163 White mothers [61.5%] vs 1064 White mothers [75.2%]; 23 mothers of other race [8.7%] vs 95 mothers of other race [6.7%]; *P* < .001). Nearly half of all mothers had a history of a psychiatric disorder (133 mothers [49.3%] of individuals born preterm and 641 mothers [45.1%] of mothers of individuals born at term; *P* = .21). Individuals born preterm were significantly more likely to have a diagnosis of BPD or asthma compared with their term-born peers (193 individuals [71.5%] vs 233 individuals [16.4%]; *P* < .001).

### Health Care Utilization

**Figure 2.**
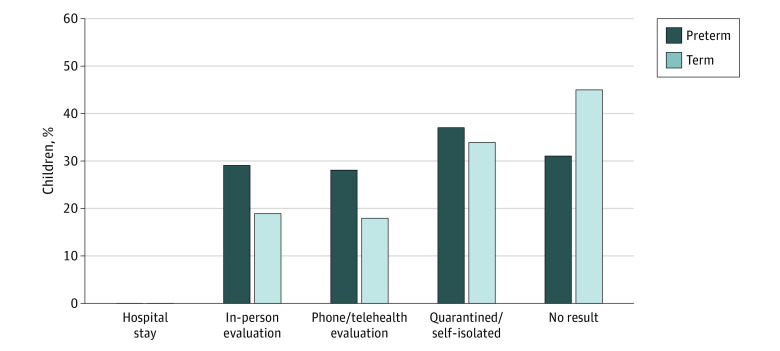
Medical Care Behavioral Patterns Due to Suspected COVID-19 Symptoms In-person evaluation includes clinic, office, urgent care, or emergency department. Phone/telehealth evaluation includes phone, email, or online.

Parents of individuals born preterm sought health care related to COVID-19 concerns for their offspring more frequently compared with parents of term-born individuals (138 individuals [51.1%] vs 478 individuals [33.6%]; *P* < .001) (Table 2). Different patterns were observed between the preterm and term groups for both in-person evaluations and phone and telehealth evaluations (Figure 2); specifically, 77 individuals (28.6%) born preterm vs 266 individuals (18.8%) born at term had in-person clinic, office, urgent care, or ED visits (*P* < .001) and 76 individuals (28.3%) vs 265 individuals (18.8%) had a phone or telehealth evaluation (*P* < .001). Approximately 35% of both groups self-isolated or quarantined. No hospitalizations were reported for either group. Additionally, group differences were seen in symptoms prompting evaluations (Table 2); for individuals born preterm, 110 (40.7%) had respiratory symptoms and 79 (29.2%) had symptoms that were more generalized (fever, headache, muscle aches), whereas for term-born individuals, 375 (26.4%) had respiratory symptoms and 339 (23.9%) had symptoms that were more generalized. In addition, fatigue was a more commonly reported COVID-19–related concern for individuals born preterm vs term (38 individuals [14.1%] vs 121 individuals [8.5%]). No individuals presented with GI symptoms. No hospitalizations due to COVID-19 symptoms were reported for either group. Approximately one-quarter of all participants (90 individuals [26.7%] born preterm vs 443 individuals [27.2%] born at term) missed health care appointments due to either parental concern of entering the practitioner office or the practitioner cancelling the appointment.

**Table 2.** Health Care Utilization Behaviors Stratified by Preterm Status

Measure	Individuals, No. (%)		*P* value
	Born preterm (<37 wk) (n = 270)	Born term (≥37 wk) (n = 1421)	
Received medical care related to concern for COVID-19 symptoms	138 (51.1)	478 (33.6)	<.001
Symptoms prompting medical evaluation			
Respiratory (cough, shortness of breath, runny nose)	110 (40.7)	375 (26.4)	<.001
Fever, headache, muscle aches	79 (29.3)	339 (23.9)	.08
Itchy red eye	14 (5.2)	72 (5.1)	>.99
Fatigue	38 (14.1)	121 (8.5)	.006
GI (diarrhea, nausea, vomiting)	0	0	NA
Loss of smell or taste	5 (1.9)	15 (1.1)	.42
How the COVID-19 outbreak affected overall health care appointments, offspring missed health care appointments	72 (26.8)	381 (27.2)	.92
Parent concerns entering health care practitioner office	39 (14.5)	216 (15.4)	.77
Practitioner cancelling appointments	51 (18.9)	227 (16.2)	.32

Abbreviation: GI, gastrointestinal.

In multivariable analysis (model 1), preterm birth was a risk factor associated with health care utilization secondary to COVID-19 concerns (aOR, 1.70; 95% CI, 1.21-2.38) (Table 3). When analyzing our primary outcome by preterm subgroups (model 2), individuals born at less than 28 weeks’ gestation had a higher odds of receiving health care (aOR, 2.15; 95% CI, 1.40-3.29) compared with term-born individuals, whereas no significant associations were observed between individuals born at 28 to 36 weeks’ gestation compared with term-born individuals (aOR, 1.32; 95% CI, 0.85-2.05) (Table 3). A maternal history of a psychiatric disorder was associated with higher health care utilization, and a diagnosis of asthma or BPD increased the odds of health care use associated with to respiratory symptoms (Table 3).

**Table 3.** Association Between Preterm Birth and Health Care Utilization Behaviors

Variable	aOR (95% CI)^a^		
	Received medical care related to concern for COVID-19	Evaluation prompted by respiratory symptoms	Evaluation prompted by any symptoms
**Model 1**			
Gestational age			
Preterm (GA <37 wk)	1.70 (1.21-2.38)	1.49 (1.05-2.12)	1.70 (1.21-2.38)
Term (GA ≥37 wk)	1 [Reference]	1 [Reference]	1 [Reference]
Maternal age^b^	1.02 (0.95-2.05)	1.01 (0.99-1.04)	1.01 (0.99-1.03)
Highest level of maternal education			
≤High school/GED	1.26 (0.86-1.84)	1.23 (0.83-1.83)	1.26 (0.86-1.84)
Some college, associate’s degree, or trade school	1.11 (0.85-1.44)	1.31 (0.99-1.73)	1.11 (0.85-1.44)
≥Bachelor’s degree	1 [Reference]	1 [Reference]	1 [Reference]
Diagnosis of offspring asthma or BPD	1.32 (1.00-1.74)	1.50 (1.13-2.00)	1.32 (1.00-1.74)
History of maternal psychiatric disorder^c^	1.44 (1.17-1.78)	1.29 (1.03-1.62)	1.44 (1.17-1.78)
Maternal race and ethnicity			
Hispanic	1.40 (0.95-2.05)	1.05 (0.70-1.59)	1.40 (0.95-2.05)
Non-Hispanic Asian	0.82 (0.42-1.61)	0.79 (0.39-1.61)	0.82 (0.42-1.61)
Non-Hispanic Black	0.98 (0.65-1.47)	0.81 (0.52-1.27)	0.98 (0.65-1.47)
Non-Hispanic White	1 [Reference]	1 [Reference]	1 [Reference]
Non-Hispanic other race^d^	1.20 (0.79-1.83)	1.24 (0.80-1.92)	1.20 (0.79-1.83)
**Model 2**			
Gestational age			
Extremely preterm (<28 wk)	2.15 (1.40-3.29)	1.78 (1.14-2.77)	2.15 (1.40-3.29)
Very, moderate, or late preterm (28-36 wk)	1.32 (0.85-2.05)	1.24 (0.78-1.95)	1.32 (0.85-2.05)
Term (≥37 wk)	1 [Reference]	1 [Reference]	1 [Reference]
Maternal age^b^	1.02 (1.00-1.04)	1.01 (0.99-1.04)	1.02 (1.00-1.04)
Highest level of maternal education			
≤High school, GED, or equivalent	1.27 (0.87-1.85)	1.24 (0.83-1.85)	1.27 (0.87-1.85)
Some college, associate’s degree, or trade school	1.10 (0.85-1.43)	1.30 (0.99-1.72)	1.10 (0.85-1.43)
≥Bachelor’s degree	1 [Reference]	1 [Reference]	1 [Reference]
Diagnosis of offspring asthma or BPD	1.29 (0.98-1.70)	1.47 (1.10-1.96)	1.29 (0.98-1.70)
History of maternal psychiatric disorder^c^	1.46 (1.18-1.80)	1.31 (1.04-1.64)	1.46 (1.18-1.80)
Maternal race and ethnicity			
Hispanic	1.39 (0.95-2.04)	1.05 (0.69-1.58)	1.39 (0.95-2.04)
Non-Hispanic Asian	0.80 (0.41-1.56)	0.78 (0.38-1.57)	0.80 (0.41-1.56)
Non-Hispanic Black	0.99 (0.66-1.49)	0.82 (0.52-1.28)	0.99 (0.66-1.49)
Non-Hispanic White	1 [Reference]	1 [Reference]	1 [Reference]
Non-Hispanic other race^d^	1.22 (0.80-1.85)	1.25 (0.81-1.94)	1.22 (0.80-1.85)

Abbreviations: aOR, adjusted odds ratio; BPD, bronchopulmonary dysplasia; GA, gestational age; GED, general educational development.

^a^ Mutually adjusted for all variables in the table, including offspring sex, singleton gestation, offspring age at COVID-19 survey completion, month/y of COVID-19 survey completion, and ECHO cohort.

^b^ Age was centered at the median, 31 years.

^c^ History of maternal psychiatric disorder was defined as a diagnosis of major depression, dysthymia, bipolar disorder, anxiety disorder, generalized anxiety disorder, specific phobia, panic disorder, obsessive compulsive disorder, social anxiety, posttraumatic stress disorder, and attention-deficit disorder/hyperactivity disorder.

^d^ The non-Hispanic other race category included those who were non-Hispanic and Alaska Native, American Indian, Asian, Native Hawaiian, or Pacific Islander, and those who identified as multiple races.

## Discussion

This cohort study found that health care use related to COVID-19–specific concerns for children and young adults born preterm was significantly higher compared with that for term-born individuals. Respiratory symptoms were the most common reason for seeking care among individuals born preterm, with approximately 40% of evaluations prompted by cough, shortness of breath, or congestion. After adjusting for a respiratory diagnosis, individuals born preterm had a higher odds of health care utilization compared with individuals born at term, which is likely driven by individuals born at less than 28 weeks’ gestation. No group differences were seen for our secondary outcome of overall health care utilization changes during the pandemic; approximately one-quarter of participants born preterm and participants born at term missed health care appointments related to parental or practitioner decision.

To our knowledge, this is the first report of differences in health care utilization during COVID-19 between individuals born preterm and at term. Studies on the use of pandemic-related pediatric health services in general are limited, yet some trends have been noted. Surges in telehealth use were seen during the early stages of the pandemic; however, significant variability has been reported. A study of 4 US academic-affiliated pediatric clinics in North Carolina and South Carolina by Brown et al^7^ reported that 15% of all visits in April 2020 were video or phone visits. While Brown et al^7^ did not include the GA of patients, there were significantly fewer visits for asthma during the pandemic compared with prepandemic (3.4% vs 2.2%). Additionally, a study by Uscher-Pines et al^32^ of 8 pediatric medical subspecialty groups in California reported that 6% to 73% of the total visits from May 2020 through April 2021 were telehealth visits.^32^ Pulmonology and neurology subspecialists reported telehealth use rates of 38.8% and 54.8%, respectively.^32^ Given that lung and brain injury is increased in the extremely preterm birth population compared with the term population, it is possible that individuals born preterm made up a greater proportion of the telehealth groups. Findings from the ECHO cohort support this hypothesis and should prompt practices to explore in more detail how individuals born preterm and with the highest risk are using practice resources. Identification of infants born preterm with high risk aligns with American Academy of Pediatric guidelines suggesting use of telehealth to ensure high-quality care for families, particularly for individuals with special health care needs.^33^

While we do not have data to distinguish illness severity prompting health care use between study groups, it could be that the individuals born preterm were more severely ill. However, we do know that no COVID-19 hospitalizations were reported in our study population, including in the extremely preterm group. This aligns with the overall low rate of COVID-19–associated hospitalizations observed among children aged younger than 18 years in the US that corresponds with our study time period.^34^

Although not the primary focus of this research, the potential role of maternal mental health was considered as a covariate. Before the pandemic, poor parental mental health has been associated with greater child ED use and contacts with primary care practitioners,^19,35,36,37^ and mothers of children born preterm report more psychiatric illnesses, including anxiety, depression, and posttraumatic stress disorder.^38,39,40,41^ We might speculate that such disorders could potentially affect parents’ anxiety about health in general, and COVID-19 illness in particular. For this ECHO study, a history of a maternal psychiatric disorder was a significant covariate associated with health care utilization among all children during the pandemic, independent of GA at birth. Given the unprecedented burden of mental distress placed on families during the pandemic, further detailed investigation could be considered.

Understanding the factors associated with overall health care use as well as symptom-related use during the COVID-19 pandemic may identify optimal practice strategies and target care, particularly during pandemic surges. Potential approaches could include COVID-19–related educational tools that address specific preterm birth–related conditions and how this may affect symptoms,^42^ refining telemedicine services tailored to individuals with special health care needs^43^ and identifying strategies that may foster parental well-being during child illness, such as coping mechanisms and resiliency.^44,45^ For this cohort, it was reassuring to see that there were no differences in overall missed health care appointments between preterm vs term-born individuals, particularly due to parental worry about entering the health care practitioner office. Recognizing the different patterns and prompts of health care use among individuals born preterm and their families during the first year of the pandemic is an important first step down the path of optimizing and tailoring care.

The ECHO Program has provided an opportunity to leverage a large sample size of 1691 individuals from 42 pediatric cohorts. Our large number of participants born preterm allowed for stratification of prematurity into subgroups and identified that infants born preterm, as well as extremely preterm, had higher rates of health care utilization related to concerns about COVID-19. Data harmonization of key variables of maternal socioeconomic status and offspring diagnosis of BPD or asthma allowed for a more nuanced exploration of factors associated with health care utilization.

### Limitations

This study has several limitations. The preterm population of this study reflects the selection criteria of some cohorts included in the ECHO Program. We recognize this study cohort was not meant to be representative of the US population in general, but rather to take advantage of a large sample size that included individuals born preterm. Our focus was contrasting the health care utilization of individuals born preterm with that of individuals born at term. We recognize that many ECHO participants were excluded due missing key data points and thus could introduce bias into the study. Findings are based on a caregiver-reported COVID-19 questionnaire and may be subject to recall bias. It is possible that participants who experienced increased COVID-19–related hardships were less likely to have returned completed COVID-19 questionnaires. These individuals may have used more or less health care based on their COVID-19 pandemic experience, potentially impacting our study findings. Rates of maternal psychiatric disorder were high in our population, we cannot exclude the possibility that mothers with psychiatric histories were more likely to complete data forms compared with mothers without such diagnoses, thus introducing selection bias. Additionally, we report the maternal psychiatric variable finding with caution, as it may reflect a variety of conditions with a range of illness severity and onset of timing and therefore limit our interpretations. Additionally, findings are COVID-19–specific, and we did not collect detailed data on non–COVID-19 health care use during the pandemic. It is possible that in general, individuals born preterm had greater health care utilization compared with term-born individuals during the pandemic. However, much of the data suggest that health care resource use by preterm-born individuals is concentrated during early childhood years,^46,47^ and for this ECHO cohort, most participants were aged 6 to 12 years.

## Conclusions

This cohort study using the ECHO Program population found independent associations between increased seeking of care secondary to COVID-19 concerns and a history of preterm birth. While evidence continues to accumulate on the effect of the COVID-19 pandemic on health care use in the pediatric population, risk factors have mostly focused on specific morbidities. The utilization of this multicohort data allows us to look beyond disease-specific risk factors and identify new subgroups of families who may be targeted by health care systems with the goal of flexible and tailored care.

## Notes

Supplement 1. **eFigure.** Time Distribution of Completed COVID-19 Questionnaires (April 2020-August 2021) **eTable.** Cohort Table

Supplement 2.
Program Collaborators for Environmental Influences on Child Health Outcomes (ECHO)

Supplement 3.
Data Sharing Statement
