# Supplementary material for: Health Care Utilization During the COVID-19 Pandemic Among Individuals Born Preterm
Source: JAMA Netw Open. 2023 Apr 28;6(4):e2310696. doi: 10.1001/jamanetworkopen.2023.10696 (PMC10148204; doi:10.1001/jamanetworkopen.2023.10696)
Supplement: Supplement 1. — eFigure. Time Distribution of Completed COVID-19 Questionnaires (April 2020-August 2021) eTable. Cohort Table [file jamanetwopen-e2310696-s001.pdf]

Supplemental Online Content

McGowan EC, McGrath M, Law A, et al; program collaborators for Environmental Influences on Child Health Outcomes (ECHO). Health care utilization during the COVID-19 pandemic among individuals born preterm. *JAMA Netw Open*. 2023;6(4):e2310696. doi:10.1001/jamanetworkopen.2023.10696

eFigure. Time Distribution of Completed COVID-19 Questionnaires (April 2020-August 2021)
eTable. Cohort Table

This supplemental material has been provided by the authors to give readers additional information about their work.

© 2023 McGowan EC et al. *JAMA Network Open.*

eFigure. Time Distribution of Completed COVID-19 Questionnaires (April 2020-August 2021)

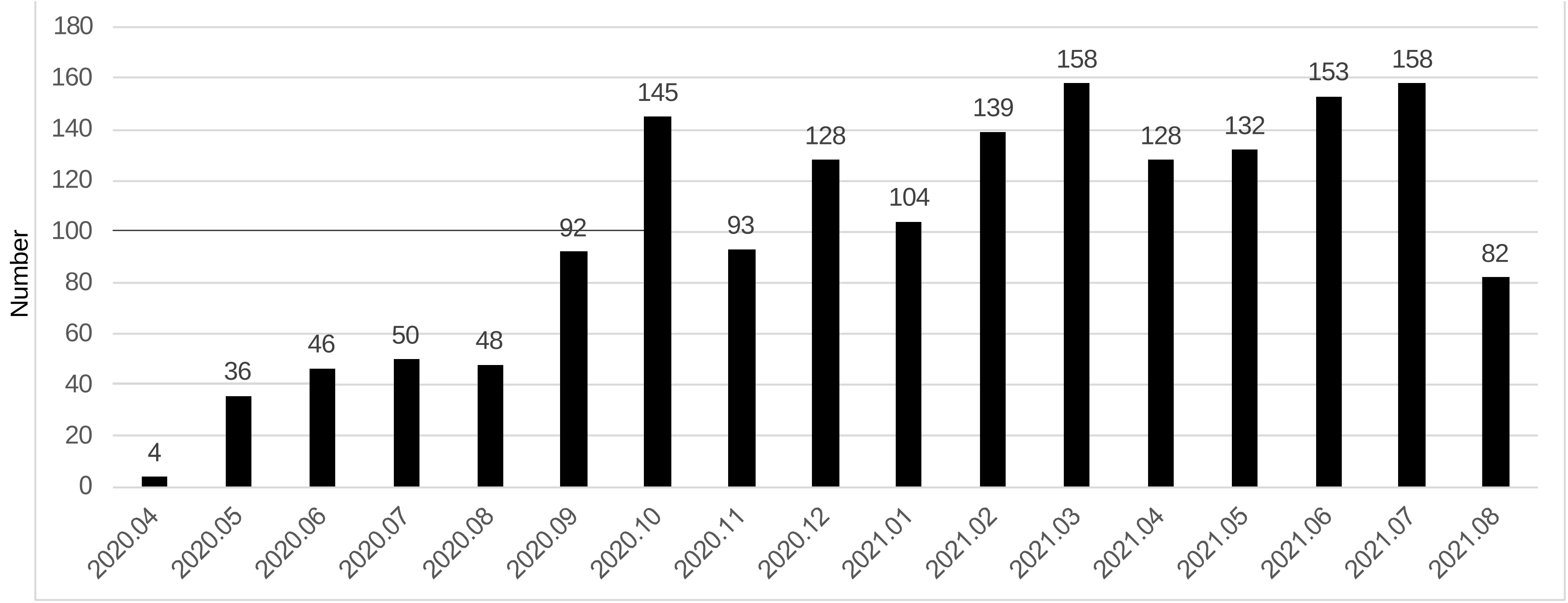

© 2023 McGowan EC et al. *JAMA Network Open.*

eTable. Cohort Table

	ECHO-WIDE COHORT	STUDY POPULATION
Sample size	(N=58283)	(N=1691)
**Gestational age at birth**		
Mean (SD)	37.9 (3.67)	37.4 (4.51)
Missing	14620 (25.1%)	1 (0.1%)
**Categorical gestational age**		
Extremely preterm (22-27 weeks)	2443 (5.59 %)	158 (9.35 %)
Very preterm (28-31 weeks)	838 (1.92 %)	67 (3.96 %)
Moderate preterm (32-33 weeks)	513 (1.17 %)	6 (0.36 %)
Late preterm (34-36 weeks)	2854 (6.54 %)	39 (2.31 %)
Early term (37-38 weeks)	10191 (23.34 %)	369 (21.83 %)
Full term (39-43 weeks)	26827 (61.44 %)	1051 (62.19 %)
Missing	14617 (25.1%)	1 (0.1%)
**Singleton gestation**		
Yes	41044 (95.15 %)	1605 (94.91 %)
Missing	15148 (26.0%)	0 (0%)
**Sex of the child at birth**		
Male	29865 (51.72 %)	900 (53.22 %)
Missing	535 (0.9%)	0 (0%)
**Child race**		
White	33881 (65.12 %)	<1270 (<76 %)
Black	8194 (15.75 %)	138 (8.22 %)
Asian	2082 (4.00 %)	37 (2.20 %)
Native Hawaiian or other Pacific Islander	187 (0.36 %)	<5 (<1 %)
American Indian or Alaska Native	1285 (2.47 %)	46 (2.74 %)
Multiple Race	5493 (10.56 %)	175 (10.42 %)
Other Race	910 (1.75 %)	16 (0.95 %)
Missing	6251 (10.7%)	12 (0.7%)
**Child Race/Ethnicity**		
Non-Hispanic White	26210 (47.84 %)	1137 (67.24 %)
Non-Hispanic Black	7330 (13.38 %)	124 (7.33 %)
Non-Hispanic Asian	1825 (3.33 %)	34 (2.01 %)
Non-Hispanic Other Race	4170 (7.61 %)	173 (10.23 %)
Hispanic	15248 (27.83 %)	223 (13.19 %)
Missing	3500 (6.0%)	0 (0%)
**Child Ethnicity**		
Non-Hispanic	39776 (72.29 %)	1468 (86.81 %)
Hispanic	15248 (27.71 %)	223 (13.19 %)
Missing	3259 (5.6%)	0 (0%)

© 2023 McGowan EC et al. *JAMA Network Open.*

**Calendar year of child birth**		
1980-2002	13036 (22.48 %)	0 (0.00 %)
2003-2005	4578 (7.90 %)	61 (3.61 %)
2006-2010	6745 (11.63 %)	282 (16.68 %)
2011-2015	18360 (31.66 %)	1194 (70.61 %)
2016-2019	12777 (22.04 %)	154 (9.11 %)
2020	1687 (2.91 %)	0 (0.00 %)
2021	799 (1.38 %)	0 (0.00 %)
Missing	301 (0.5%)	0 (0%)
**Asthma Diagnosis**		
Yes	5913 (22.40 %)	349 (20.70 %)
Missing	31889 (54.7%)	5 (0.3%)
**Age of first Asthma Diagnosis**		
Mean (SD)	7.58 (4.42)	7.14 (3.46)
Missing	54027 (92.7%)	1351 (79.9%)
**Last known Child ever had wheezing or whistling in the chest up to current visit**		
Yes	20608 (74.15 %)	1536 (91.05 %)
Missing	30491 (52.3%)	4 (0.2%)
**Last known Child had wheezing or whistling in the chest in the past 12 months**		
Yes	6044 (27.49 %)	405 (24.30 %)
Missing	36295 (62.3%)	24 (1.4%)
**Maternal age at delivery**		
Mean (SD)	29.7 (5.89)	30.4 (5.19)
Missing	11117 (19.1%)	0 (0%)
**Maternal Race/Ethnicity**		
Non-Hispanic-White	21488 (50.47 %)	1227 (73.04 %)
Non-Hispanic-Black	6644 (15.60 %)	130 (7.74 %)
Non-Hispanic-Asian	2048 (4.81 %)	47 (2.80 %)
Non-Hispanic-Other Race	2380 (5.59 %)	118 (7.02 %)
Hispanic	10019 (23.53 %)	158 (9.40 %)
Missing	15704 (26.9%)	11 (0.7%)
**Biological mother race**		
White	26782 (64.12 %)	<1310 (<80 %)
Black	7491 (17.93 %)	144 (8.62 %)
Asian	2257 (5.40 %)	47 (2.81 %)
Native Hawaiian or other Pacific Islander	195 (0.47 %)	<5 (<1 %)
American Indian or Alaska Native	1251 (2.99 %)	45 (2.69 %)
Multiple Race	2097 (5.02 %)	89 (5.33 %)
Other Race	1697 (4.06 %)	30 (1.80 %)
Missing	16513 (28.3%)	21 (1.2%)

© 2023 McGowan EC et al. *JAMA Network Open.*

**Biological mother ethnicity**		
Non-Hispanic	33549 (77.00 %)	1525 (90.61 %)
Hispanic	10019 (23.00 %)	158 (9.39 %)
Missing	14715 (25.2%)	8 (0.5%)
**History of maternal psychiatric disorder**		
Yes	5593 (37.35 %)	774 (45.77 %)
Missing	43307 (74.3%)	0 (0%)
**Maternal reported highest attained education**		
Less than high school	4751 (9.59 %)	47 (2.78 %)
High school degree, GED or equivalent	8340 (16.84 %)	120 (7.10 %)
Some college, no degree and above	36443 (73.57 %)	1524 (90.12 %)
Missing	8749 (15.0%)	0 (0%)
**Marital status**		
Married or living with a partner	27030 (77.11 %)	1286 (80.93 %)
Missing	23230 (39.9%)	102 (6.0%)

© 2023 McGowan EC et al. *JAMA Network Open.*
