# Supplementary material for: Health Care Utilization During the COVID-19 Pandemic Among Individuals Born Preterm
Source: JAMA Netw Open. 2023 Apr 28;6(4):e2310696. doi: 10.1001/jamanetworkopen.2023.10696 (PMC10148204; doi:10.1001/jamanetworkopen.2023.10696)
Supplement: Supplement 2. — Program Collaborators for Environmental Influences on Child Health Outcomes (ECHO) [file jamanetwopen-e2310696-s002.pdf]

Supplemental Online Content: Nonauthor Collaborators
*First name, last name, and suffix (if applicable) are required and will appear in PubMed.

*Group Name(s): Program Collaborators for Environmental Influences on Child Health Outcomes (ECHO)							
***First Name and Middle Initial(s)**	***Last Name**	***Suffix (eg, Jr, III)**	Academic Degrees	Institution	Location (city, state/province, country)	Role or Contribution, eg, chair, principal investigator	Group (if more than 1 Group listed in the byline) and/or Subgroup (eg, Steering Committee)
Phillip B	Smith		MD	Duke Clinical Research Institute	Durham, North Carolina, USA	ECHO Coordinating Center Prinicipal Investigator	ECHO Coordinating Center U2COD023375
Laura K	Newby		MD	Duke Clinical Research Institute	Durham, North Carolina, USA	ECHO Coordinating Center Prinicipal Investigator	ECHO Coordinating Center U2COD023375
Lisa P	Jacobson		PhD	Johns Hopkins University	Baltimore, Maryland, USA	ECHO Data Analysis Center Principal Investigator	ECHO Data Analysis Center U24D023382
Diane J	Catellier		PhD	Research Triangle Park Institute	Durham, North Carolina, USA	ECHO Data Analysis Center Principal Investigator	ECHO Data Analysis Center U24D023382
Richard C	Gershon		PhD	Northwestern University School of Medicine	Evanston, Illinois, USA	ECHO Person Reported Outcome Core Principal Investigator	ECHO Person Reported Outcome Core U24OD023319
David	Cella		PhD	Northwestern University School of Medicine	Evanston, Illinois, USA	ECHO Person Reported Outcome Core Principal Investigator	ECHO Person Reported Outcome Core U24OD023319
Susan L	Teitelbaum		PhD	Icahn School of Medicine at Mount Sinai	New York, NY, USA	ECHO Cohort Principal Investigator	ECHO Cohort UH3OD023320
Annemarie	Stroustrup		MD	Icahn School of Medicine at Mount Sinai	New York, NY, USA	ECHO Cohort Principal Investigator	ECHO Cohort UH3OD023320
Andrea L	Lampland		MD	Children’s Hospital and Clinic	Minneapolis, MN, USA	ECHO Cohort Principal Investigator	ECHO Cohort UH3OD023320
Dennis E	Mayock		MD	University of Washington	Seattle, WA, USA	ECHO Cohort Principal Investigator	ECHO Cohort UH3OD023320

Supplemental Online Content: Nonauthor Collaborators
*First name, last name, and suffix (if applicable) are required and will appear in PubMed.

***First Name and Middle Initial(s)**	***Last Name**	***Suffix (eg, Jr, III)**	Academic Degrees	Institution	Location (city, state/province, country)	Role or Contribution, eg, chair, principal investigator	Group (if more than 1 Group listed in the byline) and/or Subgroup (eg, Steering Committee)
Lisa K	Washburn		MD	Wake Forest University School of Medicine	Winston Salem, NC	ECHO Cohort Principal Investigator	ECHO Cohort UH3OD023320
Cristiane	Duarte		PhD	New York State Psychiatric Institute	New York, NY, USA	ECHO Cohort Principal Investigator	ECHO Cohort UH3OD023328
Glorisa J	Canino		PhD	University of Puerto Rico	San Jaun, PR,	ECHO Cohort Principal Investigator	ECHO Cohort UH3OD023328
Alex	Mason		PhD	University of Tennessee Health Science Center	Memphis, TN, USA	ECHO Cohort Principal Investigator	ECHO Cohort UH3OD023271
Carmen J	Marsit		PhD	Emory University	Atlanta, GA, USA	ECHO Cohort Principal Investigator	ECHO Cohort UH3OD023347
Steven L	Pastyrnak		PhD	Helen DeVos Children’s Hospital	Grand Rapids, MI, USA	ECHO Cohort Principal Investigator	ECHO Cohort UH3OD023347
Charles	Neal		MD	Kapiolani Medical Center for Women and Children	Providence, RI, USA	ECHO Cohort Principal Investigator	ECHO Cohort UH3OD023347
Jennifer B	Helderman		MD	Wake Forest University School of Medicine	Winston Salem, NC	ECHO Cohort Principal Investigator	ECHO Cohort UH3OD023347
Hyagriv	Simhan		MD	University of Pittsburgh Medical Center, Magee Women’s Hospital	Pittsburgh, PA, USA	ECHO Cohort Principal Investigator	ECHO Cohort UH3OD023349
Jean	Kerver		PhD	Michigan State University	East Lansing, MI, USA	ECHO Cohort Principal Investigator	ECHO Cohort UH3OD023285
Charles	Barone		MD	Henry Ford Health System	Detroit, MI, USA	ECHO Cohort Principal Investigator	ECHO Cohort UH3OD023285
Patricia	McKane		DVM	Michigan Department of Health and Human Services	Lansing, MI, USA	ECHO Cohort Principal Investigator	ECHO Cohort UH3OD023285
Michael R	Elliott		PhD	University of Michigan	Ann Arbor, MI, USA	ECHO Cohort Principal Investigator	ECHO Cohort UH3OD023285
Susan L	Schantz		PhD	University of Illinois, Beckman Institute	Urbana, IL, USA	ECHO Cohort Principal Investigator	ECHO Cohort UH3OD023272
Robert M	Silver		MD	University of Utah	Salt Lake City, UT, USA	ECHO Cohort Principal Investigator	ECHO Cohort UH3OD023249

Supplemental Online Content: Nonauthor Collaborators
*First name, last name, and suffix (if applicable) are required and will appear in PubMed.

***First Name and Middle Initial(s)**	***Last Name**	***Suffix (eg, Jr, III)**	Academic Degrees	Institution	Location (city, state/province, country)	Role or Contribution, eg, chair, principal investigator	Group (if more than 1 Group listed in the byline) and/or Subgroup (eg, Steering Committee)
Michelle	Bosquet-Enlow		phD	Boston Children's Hospital	Boston MA, USA	ECHO Cohort Principal Investigator	ECHO Cohort UH3OD023337
Stephanie L	Merhar		MD	Cincinnati Children's Hospital Medical Center	Cincinnati, Ohio, USA	ECHO Cohort Principal Investigator	ECHO Cohort UH3OD023320
Gloria S	Pryhuber		MD	University of Rochester Medical Center	Rochester, NY, USA	ECHO Cohort Principal Investigator	ECHO Cohort UH3OD023320
Paul E	Moore		MD	Vanderbilt Children's Hospital	Nashville, TN, USA	ECHO Cohort Principal Investigator	ECHO Cohort UH3OD023320
Andrea L	Lampland		MD	Children's Hospital and Clinic	Minneapolis, MN, USA	ECHO Cohort Principal Investigator	ECHO Cohort UH3OD023320
Rajan	Wadhawan		MD	Florida Hospital for Children	Orlando, FL, USA	ECHO Cohort Principal Investigator	ECHO Cohort UH3OD023320
Carol L	Wagner		MD	Medical University of South Carolina	Charleston, SC, USA	ECHO Cohort Principal Investigator	ECHO Cohort UH3OD023320
Lisa A	Croen		PhD	Kaiser Permanente Northern California Division of Research	Oakland, CA, USA	ECHO Cohort Principal Investigator	ECHO Cohort UH3OD023289
Lynne M	Smith		MD	Los Angeles Biomedical Research Institute at Harbour-UCLA Medical Center	Los Angeles CA, USA	ECHO Cohort Principal Investigator	ECHO Cohort UH3OD023347
Cindy	McEvoy		MD	Oregon Health and Science University	Portland, OR, USA	ECHO Cohort Principal Investigator	ECHO Cohort UH3OD023288
Robert S	Tepper		MD	Indiana University, Riley Hospital for Children	Indianapolis, IN, USA	ECHO Cohort Principal Investigator	ECHO Cohort UH3OD023288
Leonardo	Trasande		MD	New York School of Medicine	New York, NY, USA	ECHO Cohort Principal Investigator	ECHO Cohort UH3OD023305
Clement L	Ren		MD	Indiana University, Riley Hospital for Children	Indianapolis, IN, USA	ECHO Cohort Principal Investigator	ECHO Cohort UH3OD023320
Anne Marie	Reynolds		MD	University of Buffalo, Jacobson School of Medicine and Biomedical Sciences	Buffalo, NY, USA	ECHO Cohort Principal Investigator	ECHO Cohort UH3OD023320

Supplemental Online Content: Nonauthor Collaborators
*First name, last name, and suffix (if applicable) are required and will appear in PubMed.

***First Name and Middle Initial(s)**	***Last Name**	***Suffix (eg, Jr, III)**	Academic Degrees	Institution	Location (city, state/province, country)	Role or Contribution, eg, chair, principal investigator	Group (if more than 1 Group listed in the byline) and/or Subgroup (eg, Steering Committee)
Roberta	Keller		MD	University of California	San Francisco, CA, USA	ECHO Cohort Principal Investigator	ECHO Cohort UH3OD023320
Andrea J	Duncan		MD	University of Texas Health Sciences Center	Houston, TX, USA	ECHO Cohort Principal Investigator	ECHO Cohort UH3OD023320
Jonathan M	Mansbach		MD	Boston Children’s Hospital	Boston, MA, USA	ECHO Cohort Principal Investigator	ECHO Cohort UH3OD023253
Jonathan M	Spergel		MD	Children’s Hospital of Philadelphia	Philadelphia, PA, USA	ECHO Cohort Principal Investigator	ECHO Cohort UH3OD023253
Michelle D	Stevenson		MD	Norton Children’s Hospital	Louisville, KY, USA	ECHO Cohort Principal Investigator	ECHO Cohort UH3OD023253
Cindy S	Bauer		MD	Phoenix Children’s Hospital	Phoenix AZ, USA	ECHO Cohort Principal Investigator	ECHO Cohort UH3OD023253
Sean CL	Deoni		PhD	Memorial Hospital of Rhode Island	Providence RI, USA	ECHO Cohort Principal Investigator	ECHO Cohort UH3OD023313
James E	Gern		MD	University of Wisconsin	Madison WI, USA	ECHO Cohort Principal Investigator	ECHO Cohort UH3OD023282
Edward M	Zoratti		MD	Henry Ford Health System	Detroit, MI	ECHO Cohort Principal Investigator	ECHO Cohort UH3OD023282
Leonard B	Bacharier		MD	Boston Medical Center	Boston MA, USA	ECHO Cohort Principal Investigator	ECHO Cohort UH3OD023282
George T	O’Connor		MD	Boston Medical Center	Boston MA, USA	ECHO Cohort Principal Investigator	ECHO Cohort UH3OD023282
Katherine	Rivera-Spoljaric		MD	Washington University in St Louis	St Louis, MO, USA	ECHO Cohort Principal Investigator	ECHO Cohort UH3OD023282
Tina V	Hartert		MD	Vanderbilt University	Nashville TN, USA	ECHO Cohort Principal Investigator	ECHO Cohort UH3OD023282
Christine C	Johnson		PhD	Henry Ford Health System	Detroit, MI, USA	ECHO Cohort Principal Investigator	ECHO Cohort UH3OD023282
Irva	Hertz-Picciotto		MD	University of California Davis Mind Institute	Sacramento, CA, USA	ECHO Cohort Principal Investigator	ECHO Cohort UH3OD023365

Supplemental Online Content: Nonauthor Collaborators
*First name, last name, and suffix (if applicable) are required and will appear in PubMed.

*First Name and Middle Initial(s)	*Last Name	*Suffix (eg, Jr, III)	Academic Degrees	Institution	Location (city, state/province, country)	Role or Contribution, eg, chair, principal investigator	Group (if more than 1 Group listed in the byline) and/or Subgroup (eg, Steering Committee)
Scott T	Weiss		MD	Brigham and Women's Hospital	Boston, MA, USA	ECHO Cohort Principal Investigator	ECHO Cohort UH3OD023268
Robert	Zeiger		MD	Kaiser Permanente, Southern California	San Diego, CA, USA	ECHO Cohort Principal Investigator	ECHO Cohort UH3OD023268
Kristen	Lyall		ScD	Drexel Autism Institute	Philadelphia, PA, USA	ECHO Cohort Principal Investigator	ECHO Cohort UH3OD023342
Rebecca	Landa		PhD	Johns Hopkins Bloomberg School of Public Health Kennedy Krieger Institute	Baltimore, MD, USA	ECHO Cohort Principal Investigator	ECHO Cohort UH3OD023342
Sally J	Ozonoff		PhD	University of California, UC Davis Medical Center Mind Institute	Sacramento, CA, USA	ECHO Cohort Principal Investigator	ECHO Cohort UH3OD023342
Stephen R	Dager		MD	University of Washington	Seattle, WA, USA	ECHO Cohort Principal Investigator	ECHO Cohort UH3OD023342
Robert T	Schultz		PhD	Children's Hospital of Philadelphia - Center for Autism Research	Philadelphia, PA, USA	ECHO Cohort Principal Investigator	ECHO Cohort UH3OD023342
Joseph	Piven		MD	University of North Carolina at Chapel Hill	Chapel Hill, NC, USA	ECHO Cohort Principal Investigator	ECHO Cohort UH3OD023342
Heather	Volk		PhD	Johns Hopkins Bloomberg School of Public Health	Baltimore, Maryland, USA	ECHO Cohort Principal Investigator	ECHO Cohort UH3OD023342
Ruben	Vaidya		MD	Baystate Children’s Hospital	Springfield, MA, USA	ECHO Cohort Principal Investigator	ECHO Cohort UH3OD023348
Rawad	Obeid		MD	Beaumont Health Medical Center	Royal Oak, MI, USA	ECHO Cohort Principal Investigator	ECHO Cohort UH3OD023348
Caitlin	Rollins		MD	Boston Children’s Hospital	Boston, MA, USA	ECHO Cohort Principal Investigator	ECHO Cohort UH3OD023348
Kelly A	Bear		DO	East Carolina University Brody School of Medicine	Greenville, NC	ECHO Cohort Principal Investigator	ECHO Cohort UH3OD023348
Madeleine	Lenski		MS	Michigan State University College of Human Medicine	East Lansing, MI, USA	ECHO Cohort Principal Investigator	ECHO Cohort UH3OD023348
Michael E	Msall		MD	University of Chicago	Chicago IL, USA	ECHO Cohort Principal Investigator	ECHO Cohort UH3OD023348

Supplemental Online Content: Nonauthor Collaborators
*First name, last name, and suffix (if applicable) are required and will appear in PubMed.

***First Name and Middle Initial(s)**	***Last Name**	***Suffix (eg, Jr, III)**	Academic Degrees	Institution	Location (city, state/province, country)	Role or Contribution, eg, chair, principal investigator	Group (if more than 1 Group listed in the byline) and/or Subgroup (eg, Steering Committee)
Angela M	Montgomery		MD	Yale School of Medicine	New Haven, CT, USA	ECHO Cohort Principal Investigator	ECHO Cohort UH3OD023348
Tracey J	Woodruff		PhD	University of California, San Francisco	San Francisco, CA, USA	ECHO Cohort Principal Investigator	ECHO Cohort UH3OD023272
Christy A	Porucznik		PhD	University of Utah	Salt Lake City, UT, USA	ECHO Cohort Principal Investigator	ECHO Cohort UH3OD023249
Elisabeth	Conradt		PhD	University of Utah	Salt Lake City, UT, USA	ECHO Cohort Principal Investigator	ECHO Cohort UH3OD023249
R	Keller			University of Arkansas for Medical Science	Little Rock, Arkansas, USA	ECHO Cohort Principal Investigator	ECHO Cohort UH3OD023320
Carrie	Breton			University of Southern California	Los Angeles, CA, USA	ECHO Cohort Principal Investigator	ECHO Cohort UH3OD023287
Theresa	Bastain			University of Southern California	Los Angeles, CA, USA	ECHO Cohort Principal Investigator	ECHO Cohort UH3OD023287
Shohreh	Farzan			University of Southern California	Los Angeles, CA, USA	ECHO Cohort Principal Investigator	ECHO Cohort UH3OD023287
Rima	Habre			University of Southern California	Los Angeles, CA, USA	ECHO Cohort Principal Investigator	ECHO Cohort UH3OD023287
Rebecca	Schmidt			University of California, UC Davis Medical Center Mind Institute	Davis, CA, USA	ECHO Cohort Principal Investigator	ECHO Cohort UH3OD023342
Jean	Frazier			University of Massachusetts Medical School	Worcester, MA, USA	ECHO Cohort Principal Investigator	ECHO Cohort UH3OD023348
Julie	Herbstman			Columbia University Medical Center	New York, NY, USA	ECHO Cohort Principal Investigator	ECHO Cohort UH3OD023290
