# Supplementary material for: Health Care Utilization During the COVID-19 Pandemic Among Individuals Born Preterm
Source: JAMA Netw Open. 2023 Apr 28;6(4):e2310696. doi: 10.1001/jamanetworkopen.2023.10696 (PMC10148204; doi:10.1001/jamanetworkopen.2023.10696)
Supplement: Supplement 3. — Data Sharing Statement [file jamanetwopen-e2310696-s003.pdf]

Data Sharing Statement

McGowan. Health Care Utilization During the COVID-19 Pandemic Among Individuals Born Preterm. *JAMA Netw Open*. Published April 28, 2023.
doi:10.1001/jamanetworkopen.2023.10696

Data
Data available: Yes
Data types: Deidentified participant data
How to access data: De-identified data from the ECHO Program are available through NICHD's Data and Specimen Hub (DASH) (https://dash.nichd.nih.gov). DASH is a centralized resource that allows researchers to access data from various studies via a controlled-access mechanism. Researchers can now request access to these data by creating a DASH account and submitting a Data Request Form. The NICHD DASH Data Access Committee will review the request and provide a response in approximately two to three weeks. Once granted access, researchers will be able to use the data for three years. See the DASH Tutorial for more detailed information on the process (https://dash.nichd.nih.gov/resource/tutorial).
When available: With publication

Supporting Documents
Document types: None

Additional Information
Who can access the data: Researchers can now request access to these data by creating a DASH account and submitting a Data Request Form. The NICHD DASH Data Access Committee will review the request and provide a response in approximately two to three weeks.
Types of analyses: N/A
Mechanisms of data availability: Researchers can now request access to these data by creating a DASH account and submitting a Data Request Form. The NICHD DASH Data Access Committee will review the request and provide a response in approximately two to three weeks.
